# Supplementary material for: Technology Transfer of O-(2-[18F] Fluoroethyl)-L-Tyrosine (IASOglio®) Radiopharmaceutical
Source: Pharmaceuticals (Basel). 2025 May 22;18(6):769. doi: 10.3390/ph18060769 (PMC12196355; doi:10.3390/ph18060769)
Supplement: Supplementary file 1 [file pharmaceuticals-18-00769-s001.zip › pharmaceuticals-3592120-supplementary.pdf]

DeepL

Subscribe to DeepL Pro to translate larger documents.
Visit www.DeepL.com/pro for more information.

SUMMARY OF PRODUCT CHARACTERISTICS

1. NAME OF THE MEDICINE

IASOglio 2 GBq/mL, solution for injection

2. QUALITATIVE AND QUANTITATIVE COMPOSITION

1 mL contains 2 GBq of fluoroethyl-L tyrosine (^{18F}) at the date and time of calibration.

The activity per vial is then between 0.4 GBq and 40 GBq.

Fluorine-18 decays into oxygen-18 with a period of 110 min, emitting positron radiation with a maximum energy of 634 keV, followed by photon annihilation radiation of 511 keV.

Notable excipients: each mL contains 3.19 mg sodium and a maximum of 0.1 mL ethanol. For the full list of excipients, see section 6.1.

3. PHARMACEUTICAL FORM

Solution for injection.

Clear, colourless or slightly yellow solution with a pH between 4.5 and 8.5.

4. CLINICAL DATA

4.1. Therapeutic indications

This medicine is for diagnostic use only.

IASOglio is designed for positron emission tomography (PET).

PET after IASOglio injection is indicated as a diagnostic test in oncology, enabling a functional approach to pathologies, organs or tissues in which an increase in amino acid consumption is sought. The following indications in particular have been documented:

<u>Gliomas :</u>

- Characterisation of brain lesions suggestive of glioma,
- Biopsy guidance for brain lesions suggestive of glioma,
- Grade classification a glioma,
- Definition of the limits of viable tumour tissue before radiotherapy,
- Detection of viable tumour masses after treatment in cases of suspected persistence or recurrence of glioma.

4.2. Dosage and administration

<u>**Dosage**</u>

adults and the elderly

The recommended activity for an adult weighing 70 kg is 180 to 250 MBq (this activity must be adapted according to the patient's body mass, the type of camera used, PET or hybrid PET (PET-CT, PET-MRI) and the image acquisition mode). This activity must be administered by direct intravenous injection.

Renal and hepatic insufficiency

In these patients, particular attention must be paid to the activity administered, as an increase in radiation exposure is possible.

Paediatric population

Use in children and adolescents must be preceded by an analysis of the benefit/risk ratio. The activity to be administered in children or adolescents can be calculated as follows according to the recommendations of the Paediatric Working Group of the European Association of Nuclear Medicine (EANM).

When 3D PET acquisition mode, which is strongly recommended, is available, using the following formula: Administered activity [MBq] = 14 x Multiplication Factor (shown in the table below), minimum activity = 14 MBq.

If only 2D PET acquisition mode is available, using the following formula: Administered activity [MBq] = 25.9 x Multiplication factor (shown in the table below), minimum activity
= 26 MBq.

**Body mass [kg]**	Multiplication factor	**Body mass [kg]**	Multiplication factor	**Body mass [kg]**	Multiplication factor
**3**	1,00	**22**	5,29	**42**	9,14
**4**	1,14	**24**	5,71	**44**	9,57
**6**	1,71	**26**	6,14	**46**	10,00
**8**	2,14	**28**	6,43	**48**	10,29
**10**	2,71	**30**	6,86	**50**	10,71
**12**	3,14	**32**	7,29	**52-54**	11,29
**14**	3,57	**34**	7,72	**56-58**	12,00
**16**	4,00	**36**	8,00	**60-62**	12,71
**18**	4,43	**38**	8,43	**64-66**	13,43
**20**	4,86	**40**	8,86	**68**	14,00

Method of administration

For intravenous injection.

For multi-dose use.

The activity of IASOglio should be measured using an activimeter immediately prior to injection. For instructions on diluting the medicinal product administration, see section 12. For patient preparation, see section 4.4.

The injection of IASOglio must be strictly intravenous in order avoid irradiation resulting local extravasation, as well as imaging artefacts.

Image acquisition

- Dynamic acquisition of PET brain scans during the 40 minutes following injection,
- And/or simple static acquisition between 20 and 40 minutes after injection.

4.3. Contraindications

- Hypersensitivity to the active substance or to any of the excipients listed in section 6.1.
- Pregnancy (see section 4.6).

4.4. Special warnings and precautions for use

Individual justification of the benefit/risk ratio

For each patient, it must be possible to justify exposure to ionising radiation on the basis of the probable benefit. In all cases, the activity administered must be as low as reasonably achievable to obtain the diagnostic information required.

Renal insufficiency

Particular attention should be paid to the benefit/risk ratio in patients with impaired renal function, as exposure to radiation may be increased.

Paediatric population

For information on use children and adolescents, please refer the section "Use in children and adolescents".
4.2. Particular attention must be paid to the indication for examination, as the effective dose per MBq is higher than in adults (see section 11).

Preparing the patient

IASOglio should be administered to patients who have been fasting for at least 4 hours.

In order to obtain the best possible quality images and reduce exposure of the bladder to radiation, patients should be encouraged to drink sufficient fluids and empty their bladder frequently before and after the PET scan.

Interpretation of images with fluoroethyl-L tyrosine ($^{(18)\,F}$)

The images used for clinical interpretation of ^{18F} PET images must be merged with recent T2-weighted and T2/FLAIR-weighted magnetic resonance imaging (MRI) images.

On visual analysis, a qualitative assessment can be made and the target lesion can be classified as positive, when the fluoroethyl-L tyrosine (^{18F}) uptake visually exceeds the uptake activity in normal surrounding tissue, or negative, when fluoroethyl-L tyrosine (^{18F}) uptake in the target lesion is at the level of uptake in normal surrounding tissue or photopenic when uptake in the target lesion is less than fluoroethyl-L tyrosine (^{18F}) uptake in normal surrounding tissue.

To ensure intra-individual and inter-individual comparability, semi-quantitative measures of the mean and maximum fixation values of tumour activity can be calculated as ratios to a healthy-looking reference brain tissue (tumour/background ratio).
The usual threshold value for the mean value of the tumour/background fixation ratio for defining the biological volume of the tumour is greater than 1.6. The SUVmax index can also be used at 20 and 40 minutes.

The evolution of fluoroethyl-L tyrosine (^{18F}) uptake in the region of interest as a function of time can be generated from the dynamic acquisition of PET images as a time-activity curve. The shape of the time-activity curve is classified as increasing, decreasing or plateau. The maximum tumour uptake time of fluoroethyl-L tyrosine (^{18F}) can be measured. This time-activity curve can be used to differentiate glioblastoma grades (grade III-IV vs II-I).

After the examination

It is advisable to avoid close contact between the patient and young children or pregnant women for the first 12 hours after injection.

Specific warning

In the event of a hypersensitivity or anaphylactic reaction, the administration of the medicinal product must be stopped immediately and intravenous treatment started, if necessary. To enable rapid treatment in an emergency, the necessary medicines and equipment should be immediately available, in particular a tracheal intubation tube and ventilation equipment.

Depending on when the patient's injection syringe is packed, the amount of sodium contained may in some cases be greater than 1 mmol (23 mg). This must be taken into account in patients on a low sodium diet.

IASOglio contains a maximum of 10% v/v ethanol, i.e. up to 0.8 g for the maximum 10 mL dose. The resulting blood alcohol concentration in a 70 kg adult can reach 0.02 g/L (2 mg/100 mL). For the maximum adult dose, this is equivalent to 20 mL of beer or 8 mL of wine. This must be taken into account in patients suffering from alcoholism, breast-feeding women, children and patients at risk such as those suffering from liver disease or epilepsy.

Environmental precautions, see section 6.6.

The maximum volume that can be administered to a patient must not exceed 10 mL.

4.5. Interactions with other medicines and other forms interaction

interaction have been carried out.

In the case of dexamethasone treatment, a possible increase in the uptake of fluoroethyl-L tyrosine (^{18}F) by normal brain tissue must be taken into account, especially when a semi-quantitative analysis of the uptake of fluoroethyl-L tyrosine (^{18}F) is planned, for example, in the case of therapeutic follow-up or estimation of the biological volume of the tumour.

In the peri-ictal period of epilepsy, a temporary increase in the gyral uptake of fluoroethyl-L tyrosine (^{18}F) can mimic a focal lesion.

4.6. Fertility, pregnancy and breastfeeding

Women of childbearing age

When it is necessary to administer a radiopharmaceutical to a woman of childbearing age, all possibility of pregnancy must be ruled out. Any woman who has not had her period should be considered pregnant until proven otherwise. If in doubt (delayed or very irregular periods), other techniques not involving the use of ionising radiation (if available) should be considered.

Pregnancy

Examinations using IASOglio are contraindicated during pregnancy as they also involve irradiation of the foetus (see section 4.3).

Breastfeeding

Before administering a radiopharmaceutical to a woman who is breastfeeding, consideration should be given to delaying administration of the radionuclide until the mother has weaned the infant, and to choosing the most suitable product taking into account that it passes into breast milk. If administration is deemed necessary, breastfeeding should be suspended for 12 hours and the milk produced during this period should be discarded.

In addition, for radiation protection reasons, it is advisable to avoid close contact between mothers and young children for 12 hours after the injection.

Fertility

fertility studies have been carried out.

4.7. Effects ability to drive vehicles and use machines

Not applicable.

4.8. Undesirable effects

Exposure to ionising radiation may induce cancer or develop hereditary deficiencies. As the effective dose is 4 mSv when 250 MBq is administered, the maximum activity of IASOglio recommended for a 70 kg patient, these undesirable effects are only likely to occur with a very low probability.

Reporting suspected adverse reactions

It is important to report suspected adverse reactions after a medicine has been authorised. It enables the benefit/risk ratio of the medicine to be monitored on an ongoing basis. Healthcare professionals report any suspected adverse reaction via the national reporting system: Agence Nationale de Sécurité du Médicament et des Produits de Santé (ANSM) and the network of Regional Pharmacovigilance Centres - Website: https://signalement.social-sante.gouv.fr/.

4.9. Overdose

In the event of an overdose of IASOglio, the dose delivered to the patient must be reduced by increasing as much as possible the elimination of the radiopharmaceutical by forced diuresis with frequent urination. It may be useful to estimate the effective dose received by the patient.

5. PHARMACOLOGICAL PROPERTIES

5.1. Pharmacodynamic properties

Pharmacotherapeutic class: Diagnostic radiopharmaceuticals, other diagnostic radiopharmaceuticals for tumour detection, ATC code: V09IX10.

Pharmacodynamic effects

At the chemical concentrations and activities recommended for diagnostic examinations, fluoroethyl-L tyrosine (^{18F}) solution appears to have no pharmacodynamic activity.

5.2. Pharmacokinetic properties

Distribution

Pharmacokinetic studies following administration of a solution of fluoroethyl-L-tyrosine (^{18F}) show that the activity is rapidly eliminated from blood plasma.

In humans, the uptake of fluoroethyl-L-tyrosine (^{18F}) in the tissues studied peaks 15 minutes after injection, and this activity then decreases mono-exponentially with a biological half-life of 8 to 12 hours.

The plasma activity curve is bi-exponential, with biological half-lives of < 0.05 h (40%) and 14 h (60%).

Fixing to organs

Fluoroethyl-L-tyrosine (^{18F}) is actively taken up by tumour cells via the L-amino acid transport system but is neither incorporated into proteins nor rapidly degraded, resulting in a high intracellular concentration of this radiopharmaceutical.

The distribution of fluoroethyl-L-tyrosine (^{18F}) throughout the body shows that the highest activities are found in the urinary system, and to a lesser extent in the liver and salivary glands. Fluoroethyl-L-tyrosine (^{18F}) uptake in all other organs is low, and remains constant until late in the examination. No binding was observed in the bones, bile ducts or pancreas.

Elimination

Approximately 25% of the fluoroethyl-L-tyrosine (^{18F}) administered is excreted in the urine within 5 hours, corresponding to an elimination half-life of 14 hours.

It is also estimated that 99% of the fluoroethyl-L-tyrosine (^{18F}) activity administered is excreted in the urine with a biological half-life of 14 hours and that the remainder of the activity administered (1%) is eliminated via the small intestine and in the faeces.

In the urine, 60 to 70% of the activity corresponds to unmetabolised fluoroethyl-L-tyrosine (^{18F}) and 30 to 40% to different metabolic fractions. This indicates that metabolic degradation of fluoroethyl-L-tyrosine (^{18F}) occurs in the human body and that the metabolites are rapidly eliminated by the kidneys, but the clinical efficacy of fluoroethyl-L-tyrosine (^{18F}) is not affected by this metabolism.

5.3. Preclinical safety data

Toxicological studies in rats showed no mortality after a single intravenous injection of 5 mL/kg of fluoroethyl-L-tyrosine (^{18F}).

Long-term mutagenicity and carcinogenicity studies have not been performed. No studies on

reproductive function have been performed in animals.

6. PHARMACEUTICAL DATA

6.1. List of excipients

Sodium chloride

Anhydrous ethanol

Sodium ascorbate

Water for injection

6.2. Incompatibilities

This medicinal product must not be mixed with other pharmaceutical products, with the exception of those mentioned in section 12.

6.3. Shelf life

14 hours from the end of the summary.

After first use or dilution, the product should be stored for up to 8 hours, without exceeding the 14-hour expiry date from the time the synthesis ends.

6.4. Special storage precautions

This medicine does not require any special storage precautions. Do not refrigerate or freeze.

Keep original packaging.

For storage conditions after first use or after dilution, see section 6.3.

Storage must comply with national regulations on radioactive products.

6.5. Nature and contents of outer packaging

15 or 25 mL colourless glass multi-dose vial, type I of the European Pharmacopoeia, closed with a bromobutyl or chlorobutyl rubber stopper and sealed with an aluminium cap. Due to the production process, IASOglio can be supplied with a perforated rubber septum.

Presentations :

A 15 mL vial contains between 0.2 and 11 mL of solution, corresponding 0.4 to 22 GBq at calibration time.

A 25 mL vial contains between 0.2 and 20 mL of solution, corresponding 0.4 to 40 GBq at calibration time.

Multi-dose bottle.

Some presentations may not be marketed.

6.6. Special precautions for disposal and handling

General warnings

Radiopharmaceutical products must only be received, used and administered by persons authorised by the competent authorities. Their receipt, storage, use, transfer and disposal are subject to the appropriate regulations and authorisations from the competent authorities.

Radiopharmaceuticals must be prepared in such a way as to meet both radioprotection and pharmaceutical quality standards. Appropriate aseptic precautions must be taken to meet the requirements of Good Pharmaceutical Manufacturing Practice.

Do not use the product if the integrity of its packaging is compromised at any stage of its preparation.

Administration procedures must be implemented in such a way as to minimise the risk of contamination of the drug and irradiation of operators. Appropriate protective shielding is mandatory.

The administration of radiopharmaceutical products presents risks to the patient's environment due to external irradiation or contamination by urine, vomit, etc. Consequently, radiation protection measures must be taken in accordance with national regulations.

Any unused product or waste must be disposed of in accordance with current regulations.

7. MARKETING AUTHORISATION HOLDER

CURIUM AUSTRIA GMBH
GRAZER STRASSE 18
A-8071 HAUSMANNSTAETTEN Austria

8. MARKETING AUTHORISATION NUMBER(S)

- 34009 550 105 1 7, 15 mL in glass bottle
- 34009 550 105 2 4, 25 mL in glass bottle

9. DATE OF FIRST AUTHORIZATION/RENEWAL RENEWAL OF AUTHORISATION

Date of first authorisation: 23 December 2015 Date of

last renewal: 23 December 2020

10. DATE OF TEXT UPDATE

September 2024

11. DOSIMETRY

The data listed in the following table is taken from the 4th addendum to ICRP (International Commission on Radiological Protection) publication no. 53.

Organ	Absorbed dose per unit activity administered (mGy/MBq)				
	Adult	15 years old	10 years	5 years	1 year
Adrenal glands	0,014	0,017	0,026	0,042	0,077
Bladder wall	0,085	0,11	0,16	0,22	0,30
Bone surfaces	0,013	0,016	0,024	0,039	0,074
Brain	0,01	0,013	0,021	0,034	0,064
Breast	0,0095	0,012	0,018	0,03	0,057
Gall bladder	0,014	0,017	0,026	0,038	0,068
Digestive tract					
Stomach	0,013	0,016	0,024	0,038	0,069
Small intestine	0,0076	0,0094	0,014	0,02	0,032
Colon	0,011	0,013	0,021	0,032	0,054
Ascending colon	0,01	0,013	0,02	0,031	0,054
Descending colon	0,012	0,014	0,022	0,033	0,054
Heart	0,013	0,016	0,026	0,039	0,072
Kidneys	0,027	0,033	0,046	0,069	0,12
Liver	0,017	0,022	0,032	0,048	0,088
Lungs	0,014	0,02	0,028	0,042	0,081
Muscles	0,012	0,014	0,023	0,036	0,067
Oesophagus	0,012	0,015	0,023	0,036	0,069
Ovaries	0,015	0,018	0,028	0,043	0,077
Pancreas	0,014	0,018	0,027	0,043	0,078
Red bone marrow	0,013	0,016	0,024	0,038	0,072
Skin	0,009	0,011	0,018	0,029	0,055
Rate	0,013	0,016	0,024	0,040	0,073
Testes	0,012	0,016	0,025	0,038	0,070
Thymus	0,012	0,015	0,023	0,036	0,069
Thyroid	0,012	0,015	0,024	0,039	0,073
Uterus	0,017	0,021	0,034	0,051	0,086
Other fabrics	0,012	0,014	0,022	0,035	0,066
**Effective dose per unit of activity administered (mSv/MBq)**	**0,016**	**0,021**	**0,031**	**0,047**	**0,082**

The effective dose resulting the administration of a maximum activity of 250 MBq of fluoroethyl-L-tyrosine (^{18F}) is 4 mSv in a 70 kg adult.

For this activity, the radiation doses delivered to critical organs were: bladder wall: 21.25 mGy, kidneys: 6.75 mGy and liver: 4.25 mGy.

12. INSTRUCTIONS FOR THE PREPARATION OF RADIOPHARMACEUTICALS

The packaging must be checked before use and the activity measured using an activimeter.

This medicine may be diluted up to 1:20 with 9 mg/mL (0.9%) sodium chloride solution for injection.

The sample must be taken under aseptic conditions. The vial must not be used until the stopper has been disinfected. The solution must be withdrawn through the stopper using a sterile disposable syringe fitted with appropriate protection and a sterile disposable needle, or using an authorised automated dispensing system.

If the integrity of this vial is compromised, the product must not be used.

The solution should be inspected visually before use and only a clear solution with no visible particles should be used.

Detailed information on this product is available on the website of the Agence Nationale de Sécurité du Médicament et des Produits de Santé (ANSM).

CONDITIONS OF PRESCRIPTION AND DELIVERY

List I

For hospital use only.

Radiopharmaceutical products must only be used by qualified personnel. They may only be supplied to practitioners who have obtained the special authorisation provided for article R 1333-24 of the French Public Health Code.
